# Comparison of Five Rehabilitation Interventions for Acute Ischemic Stroke: A Randomized Trial

**DOI:** 10.3390/jcm14051648

**Published:** 2025-02-28

**Authors:** József Tollár, Szilvia Kóra, Petra Kós, Zoltán Vadászi, István Drotár, Péter Prukner, György Wersényi, Tamás Haidegger, Tomas Vetrovsky, Tibor Hortobágyi

**Affiliations:** 1 Somogy County Kaposi Mór Teaching Hospital, H-7400 Kaposvár, Hungarykos.petra0504@gmail.com (P.K.);; 2 Faculty of Health Sciences, Doctoral School of Health Sciences, University of Pécs, H-7621 Pécs, Hungary; 3 Digital Development Center, Széchenyi István University, H-9000 Győr, Hungary; istvan.drotar@ddc.sze.hu (I.D.);; 4 Department of Otorhinolaryngology-Head and Neck Surgery, University of Pécs Medical School, H-7621 Pécs, Hungary; 5 Hungarian Academy of Science, H-1011 Budapest, Hungary; 6 University Research and Innovation Center (EKIK), Óbuda University, Bécsi út. 96/b, H-1034 Budapest, Hungary; 7 John von Neumann Faculty of Informatics, Óbuda University, H-1034 Budapest, Hungary; 8 Faculty of Physical Education and Sport, Charles University, 162 52 Prague, Czech Republic; tomas.vetrovsky@gmail.com; 9 Department of Kinesiology, Hungarian University of Sports Science, H-1123 Budapest, Hungary; 10 Department of Sport Biology, Institute of Sport Sciences and Physical Education, University of Pécs, H-7624 Pécs, Hungary; 11 Department of Human Movement Sciences, University Medical Center Groningen, University of Groningen, 9713 AV Groningen, The Netherlands

**Keywords:** ischemic stroke, rehabilitation, exergaming, soft robot, functional outcomes

## Abstract

**Background:** Comparative efficacy of rehabilitation interventions in persons with acute ischemic stroke (PwS) is limited. This randomized trial assessed the immediate and lasting effects of five interventions on clinical and mobility outcomes in 75 PwS. **Methods:** Five days after stroke, 75 PwS were randomized into five groups: physical therapy (CON, standard care, once daily); walking with a soft robotic exoskeleton (ROB, once daily); agility exergaming once (EXE1, once daily) or twice daily (EXE2, twice daily); and combined EXE1+ROB in two daily sessions. Interventions were performed 5 days per week for 3 weeks. Outcomes were assessed at baseline, post-intervention, and after 5 weeks of detraining. **Results:** Modified Rankin Scale (primary outcome) and Barthel Index showed no changes. EXE1, EXE2, ROB, and EXE1+ROB outperformed standard care (CON) in five secondary outcomes (Berg balance scale, 10m walking speed, 6-min walk test with/without robot, standing balance), with effects sustained after 5 weeks. Dose effects (EXE1 vs. EXE2) were minimal, while EXE1+ROB showed additive effects in 6-min walk tests. **Conclusions:** These novel comparative data expand evidence-based options for therapists to design individualized rehabilitation plans for PwS. Further confirmation is needed.

## 1. Introduction

Population aging is associated with an increasing prevalence of stroke, up to 14% by age 80 [1]. As epidemiological studies are scant [2], it is difficult to ascertain the rate of walking and balance disability after a cerebrovascular accident. Some prospective cohort studies suggest that up to ~50% of stroke survivors live with movement disabilities [3]. Other studies report that up to ~95% of persons with stroke (PwS) regain walking ability during 3–6 months after a stroke [2,4]. However, it is clear that ~60% of those patients who have severe walking disability during the weeks after a stroke are likely to become highly dependent and need tailored and intensive rehabilitation options [5].

On the other hand, the statistics should be performed with an ANCOVA, dependent variable, differential score of each scale, posttest minus pretest, and a covariate of the pretest, which gives a clear impact of the improvement. Stroke rehabilitation and the structural rehabilitation of the spine and posture intersect through their shared focus on restoring biomechanical function, improving motor control, and addressing postural impairments. Rehabilitation techniques nowadays heavily rely on advanced technologies, such as robotics and Virtual Reality training, to enhance these outcomes. Alongside these innovations, evidence-based approaches like mirror therapy (MT) and cognitive therapeutic exercise (CTE) have demonstrated significant promise in improving motor and sensory recovery. When combined with task-oriented training, these interventions have been shown to produce substantial improvements in upper limb functionality, sensitivity, and range of motion. While no significant differences have been observed between MT and CTE in effectiveness, their integration into rehabilitation plans underscores the importance of personalized strategies to optimize recovery outcomes in stroke patients [6,7,8,9,10,11,12].

There is indeed evidence that a single type of exercise intervention can enhance standard rehabilitation and improve clinical symptoms, walking capacity, and balance. For example, moderate certainty evidence suggests that high-intensity aerobic training (n = 47 studies) improves fitness by 3.2 mL kg^−1^ min^−1^ indexed by oxygen consumption and walking speed by 0.34 m s^−1^ compared with lower-intensity or usual care exercise (3.0 mL kg^−1^ min^−1^, 0.13 m s^−1^) [13]. Game-based vs. standard care comparisons of interventions (n = 22 studies), especially at high intensity [14,15], also improved measures of mobility and balance (*p* ≤ 0.010) [16]. Robot-assisted gait re-training is evolving as a new therapeutic approach to rehabilitation of walking and balance dysfunctions, but at this time, its superiority over standard care is unclear (n = 27 studies) [17] and needs further confirmation [18,19]. Virtually all of the 111 individual studies reviewed [16,17,18,19] compared one specific intervention type with standard care, providing no insights into the comparative effectiveness of multiple interventions determined within one randomized trial. Such a design would reduce between-study variations and allow for the determination of comparative effectiveness with higher fidelity, including the lasting effects of the interventions following a period of detraining [14,15]. Therefore, the purpose of the present randomized trial was to compare the immediate and lasting effects of five interventions on the clinical and mobility outcomes of PwS. We addressed the following questions:

1. Are innovative methods (EXE, ROB) better than standard care, and is either superior over the others?
2. Does increasing the daily dose (EXE2 vs. EXE1 daily) improve the outcomes in a dose-dependent manner?
3. If so, is combining two modalities (EXE1+ROB) more effective than one modality (EXE1 or ROB) or doubling one modality (EXE2)?

## 2. Materials and Methods

### 2.1. Study Design and Participants

**Figure 1:**
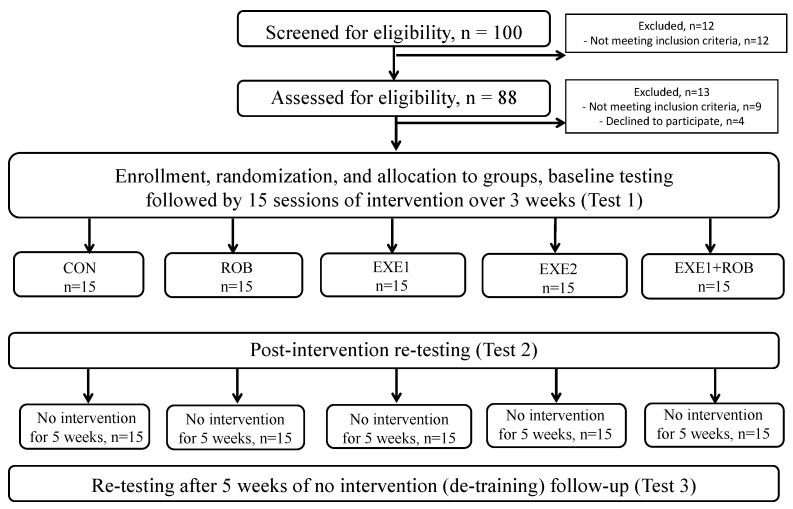
Study design.

This was an assessor-blinded, parallel 5-group, and detraining randomized clinical trial. A physical therapist not involved in this trial performed the concealed randomization by drawing a colored ribbon from a covered box and attaching one ribbon to each patient’s folder. A neurologist identified and examined each patient for admission to this study. All PwS were in the sub-acute state and stayed in the hospital’s in-patient care for five days after the stroke. They were then discharged and visited the hospital’s rehabilitation center as outpatients. Inclusion criteria: CT- or MRI-diagnosed first-ever ischemic stroke, 5 days after stroke; mobility and postural limitation with a modified Rankin Scale (mRS) score ≥ 2. Exclusion criteria: a history of multiple strokes, resting systolic blood pressure (BP) < 120 or >160 mm Hg, orthostatic hypotension, carotid artery stenosis, severe heart disease, hemophilia, traumatic brain injury, seizure disorder, uncontrolled diabetes, abnormal EEG, Mini Mental State Examination (MMSE) score < 22, an abnormal blood panel, use of sedatives, irregular medication schedule, serious aphasia, serious visual or hearing impairments, serious sensory dysfunction, serious orthopedic problems, neurological conditions affecting motor function (Parkinson’s, multiple sclerosis, multiple system atrophy, Guillain–Barré syndrome), alcoholism, recreational drug use, inability to walk a minimum of 100 m with or without a walking aid in six minutes, Berg Balance Scale (BBS) score ≤ 32, Barthel Index (BI) score ≤ 70, inability to understand verbal instructions or prompts from a TV screen or current participation in a self-directed or formal group exercise program other than standard physical therapy. Figure 1 shows the study design. Participants signed a written informed consent. The Institutional Research Ethics Committee approved the study protocol (IKEB2023/01), which was registered as a clinical trial (NCT05985889).

### 2.2. Interventions and Detraining

Fifteen eligible PwS (n = 75) were randomized to one of five intervention groups after baseline measurements (Test 1): physical therapy once a day (standard care control, CON), walking with an exoskeleton robot once a day (ROB), exergaming once a day (EXE1), exergaming twice a day (EXE2), and exergaming combined with robot training once a day (EXE1+ROB). Each intervention lasted three weeks. Participants attended either one session daily (EXE1, ROB, EXE1+ROB, CON) or two sessions daily (EXE2) every weekday, amounting to 5 or 10 sessions per week (Figure 1). After each session, PwS received 20 min of medical massage for the lower extremities. Patients made up sessions missed due to illness or social commitments. The interventions were delivered by physical therapists who were not involved in testing.

Two to three days after the last intervention session, the measurements were re-administered (Test 2). A five-week-long detraining followed, and after this follow-up period, this study was concluded with the measurements re-administered again (Test 3). During the 5-week-long detraining, all therapy sessions were stopped. We asked PwS to record their symptoms in a log that was checked by therapists daily. We asked PwS not to change diet, medication, or physical activity habits during this study. The log data were not analyzed systematically and are not reported here. The trial was conducted in the rehabilitation gym of a state hospital. Before the start of this study, all PwS were enrolled in standard physical therapy provided by social insurance, and all those allocated to CON continued this care as one of the interventions, but all others stopped receiving this care.

*Physical Therapy (CON):* Participants received 30 min of group exercise (sitting) and 30 min of individual therapy focused on walking and balance exercises at local clinics.

*Agility Exergaming (EXE1, EXE2):* Participants engaged in high-intensity, non-immersive exergaming (perceived exertion 14–16/20) and agility training. Exergaming (25 min) utilized Xbox 360 modules (e.g., Reflex Ridge, Space Pop, Just Dance) to enhance reflexive and coordinated responses. Agility training (25 min) included walking on variable surfaces, manipulating objects, and reacting to sensory cues to improve balance, coordination, and postural control. EXE1 performed these routines once daily (1 h), while EXE2 completed them twice daily (2 × 1 h).

*Robot-Assisted Walking (ROB):* Sessions included warm-up, familiarization, and guided walking with a robotic device. Activities involved hallway walking, slalom walking, and alternating intensity walking, with a heart rate target of 110–130 bpm. Each session concluded with stretching. Total duration: ~40 min.

*Combination Therapy (EXE1+ROB):* Participants combined morning exergaming (1 h) with afternoon robot-assisted walking (1 h), totaling 2 h daily.

*Walking with the robot (ROB):* PwS were equipped with the robot and a heart rate monitor in ~5 min. A warm-up of 5 min followed and involved passive movements of lower extremity joints. Next, PwS performed 5 min of familiarization walking with the robot to learn to rely on the robot while walking. PwS, escorted by a therapist, walked for 10 min at a heart rate of 110–130 b min^−1^ along a 100-m-long hallway. They also performed 10 min of slalom walking around obstacles placed ~1 m apart over a 20-m-long course with 1 min of rest every 2 min. The aim was to walk as rapidly and safely as possible. Finally, PwS walked for 6 min, alternating high for 50 m vs. low for 50 m intensity walking. PwS, at the end of the session, sat down, and the robot was taken off in about 5 min. The sessions ended with 10 min of stretching.

*Combination of exergaming and walking with the robot (EXE1+ROB):* PwS performed EXE in the morning (1 h) and ROB in the afternoon (1 h) for a total of 2 h.

### 2.3. Outcomes

Primary and secondary outcomes were measured at Tests 1, 2, and 3, by the same examiners who were blinded to intervention allocation. The testing order was standardized among PwS and testing sessions.

*Primary outcome*: mRS measures independence in activities of daily living and discriminates among less severe levels of stroke disability. mRS is a reliable and valid measure that is sensitive to change over time. A change in one unit in mRS is considered clinically meaningful [20,21]. A physical therapist specifically trained in mRS administered this test.

*Secondary outcomes*: These tests are reliable, valid, and sensitive to change over time at PwS. BI measures activities of daily living performance [22]. BBS measures fall risk [23], and 6 MWT measures fitness and walking capacity [24]. After familiarization, we measured static balance by COP sway velocity while standing on a force platform [25]. Sway velocity was averaged across four standing conditions: wide and narrow stance with eyes open and closed, using 1, 20-s-long trial per condition.

We also measured the distance PwS walked during another 6 MWT while wearing the robot (6 MWT robot, ~5 kg). We used the ReStore Soft Exo Suit, an exoskeleton robot designed for post-stroke rehabilitation of gait (ReWalk model, Fototronic Kft., Budapest, Hungary) [26,27,28]. It took 5 min to outfit PwS with the robot. On the first occasion, PwS were familiarized with the robot and a therapist passively moved the hip, knee, and ankle joints through the range of motion for 5 min. The robot comprises battery-powered motors and a control unit housed in an actuator pack at the waist. One cable from the motors is attached to the proximal part of the functional textile covering the calf. A second cable is attached distally to the shoe insole. None of the PwS needed an additional ankle–foot orthosis to prevent ankle inversion. Inertial sensors in the shoes measure gait events. The control unit recorded the movement pattern of the non-paretic side. Load cells on each cable were set at the level of ankle motion assistance. Through the cables, ankle plantarflexion and dorsiflexion of the involved paretic ankle could be aided. For each patient, the control unit could adjust the magnitude of ankle motion in real time via Bluetooth. Walking balance was displayed on the hand-held control unit’s touchscreen. In this unit, therapists could select the level of ankle motion assistance and monitor patient performance and progress.

Resting heart rate (HR, Polar model RS800CX HR watch, Polar Electro Co. Ltd., Kempele, Finland) and resting blood pressure (BP, Omron M7 Intelli IT, OMRON Healthcare UK Ltd., Milton Keynes, UK) were measured in sitting 5 min before and after each session and peak HR was measured during each exercise session. During the first visit, PwS became familiar with exergaming by watching and performing modules and completing questionnaires. The 2nd visit included the measurements of body height and mass, COP, and 6 MWTs. HR was measured during the 1 h interventions as well, and peak averaged HR was computed from the 5 highest values. The rate of perceived exertion during each session was recorded on a 20-point Borg scale.

### 2.4. Outcome Measures

The primary outcome parameter was the predicted probability (PP) of passing the hand scan based on the HH education group. Hand scans were categorized as “passed” if a minimum coverage of 95% of each hand surface (including the palm and dorsum of both the left and right hands) was achieved. Secondary outcome parameters were the identification of the most frequently missed areas of hand antisepsis as well as the parents’ acceptability of the video.

### 2.5. Statistical Analysis

Data were reported as mean ± SD, median and interquartile range, or 95% confidence interval. We used ordinal regression of a proportional-odds model to determine the effects of Group (interventions) on mRS, the primary outcome. We analyzed the secondary outcomes using linear regression to test the effects of Group (interventions) at post-intervention and following detraining. Only for those variables that were significant at post-intervention we explored the lasting effects of the interventions after 5 weeks of detraining. In case of a significant effect, simultaneous tests for general linear hypotheses were performed as post-hoc analyses to explore between-group differences. *p*-values and confidence intervals were adjusted for multiple comparisons using the single-step method. To determine the effects of exercise duration dose (1 h vs. 2 h), ‘duration’ was created as a new variable, and exercise mode was used as a covariate. All models were adjusted for age, sex, and baseline values. HR, rate of perceived exertion, and BP data were analyzed with appropriately modeled analysis of variance with repeated measures and the effects characterized by partial eta squared, pη^2^. Within group effects over time were further characterized by Cohen’s effect size, d. The level of significance was set at *p* < 0.05. The analyses were performed in R (version 4.3.1, The R Foundation for Statistical Computing, Vienna, Austria) using the ‘lme4’, ‘multcomp’, and ‘ordinal’ packages and in SPSS version 29.

## 3. Results

### 3.1. Patient Characteristics, Baseline Data

Table 1 shows the descriptive data of the 75 PwS who were admitted to this study 5 days (±0.00) after an ischemic stroke in the left (53%) or right hemisphere (28%) or in the cerebellum (19%). Age, height, mass, and body mass index, respectively, were 66.0 y (±4.82, range, 54–77), 174.0 cm (±6.23, 157–190), 75.7 kg (±8.00, 57–97), and 25.0 kg m^2^ (±2.55, 19–32). At 75 PwS, resting heart rate, systolic and diastolic blood pressures were 84.0 b min^−1^ (±5.09), 130.1 mm Hg (8.90), and 89.5 mm Hg (8.93), respectively. From the 16 co-morbidities, hypertension (27%), ischemic heart disease (16%), and type 2 diabetes (15%) were the most frequent ones, followed by rheumatoid arthritis, osteoporosis, atherosclerosis, COPD, colitis ulcerosa, hypercholesteremia, balance dysfunction, depression, benign paroxysmal positional vertigo, epilepsy, inflammatory bowel disease, thyroid dysfunction, and atrial fibrillation. From the 75 PwS, 48% smoked, 20% consumed 1–3 drinks daily, and 91% took medications. The median mRS score was 4.0 (IQR: 2.00 to 4.00).

### 3.2. Intervention Effects

*Primary outcome:* There was no effect of Group on mRS, the primary outcome (χ2 = 5.4, *p* = 0.246). Table 1 shows the data for the intervention and detraining effects on the primary and secondary outcomes at the three measurement points.

*Secondary outcomes:* There was no effect of the Group on the Barthel Index (F = 1.1, *p* = 0.353). The Group main effect suggested that the 5 groups differed after the intervention in the Berg balance scale scores (F = 24.2), 6 MWT (F = 29.4), 6 MWT robot (F = 30.6), 10 m maximal walking speed (F = 6.7), and COP sway velocity (F = 7.5, all *p* < 0.001).

Table 2 shows the post-hoc analyses for the significant Group main effects, i.e., the between-group differences in values after the intervention. The intervention-induced improvements in the 4 groups vs. CON were greater in the Berg balance scale scores (range of d = 1.3–4.7), 6 MWT (d = 2.0–3.6), 6 MWT robot (d = 1.3–3.2), 10 m walking speed (d = 0.9–2.5), all *p* ≤ 0.004, except for EXE1 vs. CON (*p* > 0.05) in the 10 m walking speed. Furthermore, EXE1 vs. ROB improved the distance walked in 6 MWT by 88 m or 62% (d = 1.4, *p* = 0.006) but did not affect other outcomes. In addition, EXE2 vs. EXE1 improved the Berg balance scale scores ~15% more (d = 1.4, *p* = 0.001), but there was no dose effect in other outcomes. Finally, the EXE+ROB vs. EXE2 comparison revealed a more favorable effect in 6 MWT with and without the robot (d = 1.1–2.2, *p* ≤ 0.002) but did not in other outcomes.

### 3.3. Detraining Effects

The Group main effect suggested that the 5 groups differed after detraining in the Berg balance scale scores (F = 22.0), 6 MWT (F = 28.6), 6 MWT robot (F = 13.5), 10 m maximal walking speed (F = 4.8), and COP sway velocity (F = 7.7, all *p* < 0.002).

Table 3 shows the post-hoc analyses for the significant Group main effects, i.e., the between-group differences in the values after detraining. Differences between the 4 intervention groups vs. CON were still present after 5 weeks of detraining (all *p* < 0.001) except for EXE1 and EX2 comparisons for the 10 m walking speed test. However, none of the other between-group comparisons were significant after detraining in 10 m walking speed and COP sway velocity (all *p* > 0.05). The EXE2 vs. EXE1 comparison was not significant for any of the outcomes. The EXE1+ROB combined training produced superior lasting effects in the Berg balance scores (vs. EXE2) and 6 MWT with (vs. EXE1 and vs. EXE2) and without (vs. ROB) the robot.

### 3.4. Dose Effects

We examined the dose effects of 1 h vs. 2 h on outcomes. Two vs. one h intervention per day improved the Berg balance performance (effect estimate = 5.9 points, ±SE: 1.0, *p* = 0.001) and 6 MWT (effect estimate: 43.2 m ±15.3, *p* = 0.006). These effects were sustained after 5 weeks of detraining (Berg balance test: effect estimate = 7.0 points ± 1.3, *p* = 0.001; 6 MWT: effect estimate = 57.1 m ±17.4, *p* = 0.002). There were no such dose effects on the Barthel index, 6 MWT robot, 10 m walking speed, and COP sway velocity (all *p* > 0.05).

### 3.5. Specificity of Walking with the Robot

The data in Table 1 indicate no significant baseline differences (*p* > 0.05) among the five groups in key performance measures, such as the 6-min walk test (6 MWT) and robot-assisted 6-min walk test (6 MWT robot). However, participants consistently covered longer distances using the robotic system compared to conventional walking, as demonstrated by a ~116 m advantage at baseline (*p* = 0.001, d = 3.7). This underscores the potential benefits of robot-assisted walking in stroke rehabilitation, even at the start. Post-intervention, the 6 MWT distances improved substantially in all groups, with the robot-assisted 6MWT showing a greater improvement (50.2 m) compared to the conventional 6 MWT. Notably, the EXE1+ROB group exhibited the most significant gains in robot-assisted walking, increasing by 174.7 m (d = 4.38) after the intervention. Importantly, this improvement persisted even after five weeks of detraining, with an additional gain of 186.0 m, demonstrating the sustainability of this combined intervention’s effects. In contrast, the control group (CON) showed minimal improvement in both walking measures, with only a 4.0 m increase post-intervention and a modest gain of 26.0 m after detraining. These findings highlight the limited efficacy of standard care in producing significant mobility gains, further emphasizing the advantage of intensive interventions like EXE1+ROB. The results suggest that combining high-intensity agility training with robot-assisted walking yields superior outcomes, particularly in walking distance and sustainability, compared to standard care or isolated interventions.

### 3.6. Rate of Perceived Exertion, Heart Rate, and Blood Pressure Data

Heart rate measured for 1 min in seated rest 5 min before each of the 15 sessions remained similar across groups (84.0 ± 5.09 bpm) but increased by approxi-mately 2 bpm (d = 0.37) to 86.1 bpm (±6.30, *p* = 0.001) after the 15 sessions. Systolic blood pressure decreased from 130.1 mmHg (±8.90) to 126.7 mmHg (±7.69, *p* = 0.001), while diastolic blood pressure dropped from 89.5 mmHg (±8.93) to 84.6 mmHg (±5.67), showing a reduction of approximately 4.9 mmHg (*p* = 0.001, d = 0.63). Perceived exer-tion was highest in EXE1+ROB (14.8 ± 1.07) and lowest in EXE1 (12.5 ± 1.12, Group effect: *p* < 0.001). Peak heart rate was highest in EXE2 (137.0 ± 6.69) and lowest in CON (124.8 ± 6.80, Group effect: *p* < 0.001). Exertion and peak heart rate values fluctuated over the 15 sessions but did not show significant overall changes

## 4. Discussion

We compared the immediate and lasting effects of five interventions on clinical and mobility outcomes in persons with stroke (PwS). The patients included in our study had experienced a stroke five days prior to the initiation of these interventions and presented with very poor walking capacity. Ischemic stroke remains one of the leading causes of disability and mortality worldwide, disproportionately affecting individuals with underlying conditions such as hypertension, diabetes, and cardiovascular disease. These comorbidities not only elevate the risk of stroke but also hinder recovery, impacting mobility, balance, and overall functional independence. Recent research has emphasized the role of socioeconomic and racial disparities in stroke incidence and recovery outcomes, with certain populations facing higher rates of mortality, greater levels of disability, and reduced access to advanced rehabilitation interventions. Global health challenges such as the COVID-19 pandemic have further complicated stroke management. COVID-19 has been associated with a hypercoagulable state, severe inflammation, and endothelial dysfunction, all of which exacerbate the risk of ischemic stroke. Moreover, the interplay between these mechanisms and pre-existing conditions intensifies the complexity of recovery, particularly in patients with overlapping cardiovascular or metabolic disorders. These findings underscore the need for innovative rehabilitation approaches that address both the systemic and neurological consequences of stroke to improve recovery trajectories and reduce long-term disability. We observed that none of the interventions significantly improved the primary outcome, the modified Rankin Scale (mRS), or the Barthel Index as a secondary outcome. However, each of the innovative interventions—ranging from exergaming (EXE1 and EXE2) to robot-assisted walking (ROB) and their combination (EXE1+ROB)—produced superior and lasting effects compared to conventional therapy (CON) in most secondary outcomes. Notably, the EXE1 intervention demonstrated greater improvement in the 6-min walk test (6 MWT) without a robot compared to ROB. Despite these advancements, increasing the daily dose of one specific intervention, as in EXE2 compared to EXE1, did not yield additional improvements in secondary outcomes, apart from a significant increase in the Berg Balance Scale (BBS). The combination of exergaming and robot-assisted walking (EXE1+ROB) was found to produce additive or synergistic effects, particularly in 6 MWT outcomes. This combined intervention proved more effective than doubling the frequency of exergaming alone, as seen in EXE2, but only in two of the five secondary outcomes examined. These results highlight the importance of multimodal and carefully tailored rehabilitation strategies to maximize functional recovery in stroke patients. By integrating approaches that address both physical and cognitive deficits, we can better meet the complex needs of patients recovering from acute ischemic stroke [29].

### 4.1. Sample Characteristics at Baseline

The current sample of male (n = 43) and female (n = 32) PwS was of normal body mass, BMI, and blood pressure (130/81 mm Hg), had a typical distribution of smokers, alcohol consumers, and medication-controlled hypertensive individuals with a somewhat high resting heart rate (84 b min^−1^) (Table 1). Five days after stroke at baseline, the mRS and the Barthel Index scores suggested mild levels of ADL-specific disability (Table 1). While a rapid walking speed of 0.9 m s^−1^ measured over a 10-min distance reflects mild levels of mobility disability [30], the ~120 m 6 MWT performance suggests severely impaired walking capacity. From the PwS we have examined in our previous studies, the ~120 m is the shortest distance yet covered by PwS [14,15], but also Parkinson’s disease (232 m), multiple sclerosis (334 m), or healthy older adults (529 m) [6,29,31,32]. According to recommendations [33], we selected PwS in a subacute state because such individuals are in the most urgent need for recovery and also respond favorably to intensive mobility interventions [14,15,34].

### 4.2. Are Innovative Methods (EXE, ROB) Better than Standard Care, and Is Either Superior over the Others?

Table 2 provides consistent evidence for the superior effects of EXE and ROB over standard care (CON) in the BBS, 6 MWT with and without the robot, 10 m walking speed (only after ROB), and static balance as measured by COP. We [6,14,15,32,35,36,37] and others [38,39,40] have extensively documented the higher effectiveness of EXE vs. CON in several patient groups. While biomechanically, neurophysiologically, and through imaging not yet examined [16], the EXE’s effectiveness might lie in concurrently targeting and improving sensorimotor, proprioceptive, and balance functions underlying gait and postural control. Our ROB data also suggest high efficacy for improving gait and balance. Unlike EXE, ROB is highly specific to gait function and acts through motor re-learning of (alternating) stepping. While ROB is still evolving as a new therapeutic approach to rehabilitation of walking and balance dysfunctions at PwS [16,17,18,19], our data align with some of the 27 studies that reported its superiority over standard care [10].

The EXE vs. ROB data provide a between-intervention comparison for the first time [16,17,18,19]. We observed no differences in the effects between ROB vs. EXE1 in four of five secondary outcomes. Unexpectedly, 6 MWT performance without the robot was 56 m worse in ROB vs. EXE, underscoring again the importance of EXE targeting multiple systems underlying walking mobility (Table 2). This observation is supported by the data showing no difference between EXE1 and ROB in improving 6 MWT while wearing the robot. These comparative effectiveness data suggest that acute PwS under the current experimental conditions could be expected to respond favorably to low-cost, high-intensity EXE designed to target multi-system dysfunctions than a higher-cost, gait-specific ROB intervention. Table 3 shows that PwS successfully maintained the EXE1 and ROB-induced improvements in several secondary outcomes after 5 weeks of detraining. These maintenance effects were selectively higher only in the BBS and 6 MWTrobot after ROB vs. EXE1.

### 4.3. Does Increasing the Daily Dose (EXE2 vs. EXE1) Improve the Outcomes in a Dose-Dependent Manner?

Current recommendations for exercise rehabilitation of subacute PwS promote moderate-intensity exercises [41,42]. However, high exercise intensity for motor rehabilitation has been promoted for Parkinson’s disease [6,36,43], multiple sclerosis [44], spinal cord injury [45], PwS [14,15,46], and even after ischemic heart attacks [47]. One possibility to achieve high exercise intensity is increasing its frequency, i.e., consecutive daily sessions or multiple sessions within a day [48]. Another marker is HR > 60% of the age-predicted maximum and RPE higher than 15/20. These interventions met both criteria, as peak HR was ~90% of the age-predicted maximum (137/154 b min^−1^), and EXE2 was also exercised twice a day. Yet Table 2 and Table 3 show fundamentally no frequency dose-effects, as improvements in 4 of 5 secondary outcomes did not differ between EXE1 vs. EXE2 after the interventions and detraining. The current results partially agree with a previous study reporting that in 5 of 6 outcomes, the EXE2 group improved the most compared with the EXE1 and CON groups [14]. PwS in the current sample started to exercise sooner, only five days after stroke, and had even greater mobility impairments than PwS in that previous study [14]. We observed no negative effects of EXE2 on outcomes, suggesting that PwS tolerated well the twice daily interventions, confirming our previous observations.

### 4.4. Is Combining Two Interventions More Effective than One Intervention?

We examined the idea that combining two intervention modalities might have an additive, synergistic, or perhaps an interference effect on outcomes. The bottom three rows of Table 2 and Table 3 show the relevant data, revealing additive or synergistic effects fundamentally only in the two 6 MWT outcomes, which lasted after 5 weeks of detraining. The potentiating effect was most likely related to the robot aiding the cyclical walking movement and also to the very low walking capacity at baseline. Some support for this argument comes from the r = 0.57 (*p* = 0.001, n = 75) correlation between baseline 6 MWT performance while wearing the robot and the increases in this performance. This association was lower (r = 0.22, *p* = 0.058, n = 75) for the non-robot 6 MWT. Because our PwS had very poor walking capacity as indexed by the 6 MWT performance (~123 m), we note that donning the soft exoskeleton robot already helped PwS walk ~116 m longer (d = 3.7, Table 1), and this performance increased to ~274 m without and to ~324 m with the robot (Table 2). These are important data because such walking distances can already serve as a training stimulus for cardiovascular function and walking capacity.

Combining two interventions (i.e., EXE1+ROB) seemed to provide inconsistent benefits over the effects produced by doubling one intervention (EXE2). For example, the BBS scores decreased by ~4 points, 10 m walking speed and standing sway (COP) did not differ, but the two outcomes of the 6 MWT both benefited after EXE1+ROB vs. EXE2.

## 5. Limitations

While we instructed PwS not to change their diet or physical activity, we did not systematically monitor these factors, which could have influenced outcomes. Considering the very low walking capacity and frequent reports of fatigue among PwS, it is a limitation that we did not measure fatigue during the assessments. Similarly, we did not assess premorbid physical activity or cognitive reserve (CR), both of which are known to influence rehabilitation outcomes. Recent studies have highlighted that higher CR, influenced by factors like education and engagement in cognitively stimulating activities, is associated with better functional recovery post-stroke. Premorbid physical activity also plays a role in mitigating stroke severity and enhancing rehabilitation outcomes, suggesting these factors should be evaluated in future research. While our study demonstrated the effectiveness of exoskeleton-assisted gait training, we did not measure neurophysiological changes such as muscle activation patterns or prefrontal cortex activity. Evidence from recent studies suggests that exoskeletons can enhance cortical reorganization and neuroplasticity during gait training. Understanding these mechanisms could provide critical insights into optimizing interventions for stroke survivors. Aspects such as user preferences, acceptance, and sustainability of exoskeleton use remain unexplored and warrant further investigation to enhance the long-term viability of robotic rehabilitation technologies. In conclusion, while needing further confirmation, these novel comparative efficacy data increase the evidenced-based intervention options therapists can choose from to design individually tailored rehabilitation of PwS [7,8,9,10,11,12].

## 6. Conclusions

In conclusion, while these findings require further confirmation, the comparative effectiveness data provided by this study expand the evidence-based options available to therapists for designing individually tailored rehabilitation programs for PwS. By incorporating these novel interventions, therapists can better address the diverse needs of stroke patients, potentially enhancing recovery outcomes and quality of life [49,50,51,52,53,54]. It is evident that further research and multi-center investigations are required to establish international protocols and update the current guidelines for more efficient therapies.

## Figures and Tables

**Table 1:** Frequency, medical, and baseline data for stroke patients included in this study.

	CON		ROB		EXE1		EXE2		EXE1+ROB	
	$\bar{x}$	±SD,%	$\bar{x}$	±SD,%	$\bar{x}$	±SD,%	$\bar{x}$	±SD,%	$\bar{x}$	±SD,%
Age, y	64.7	5.31	65.0	5.10	66.9	4.61	65.5	4.66	67.9	4.15
Height, cm	173.3	6.41	174.1	5.58	174.6	7.00	172.2	7.16	175.9	4.92
Mass, kg	73.3	7.72	75.8	6.73	76.7	10.17	71.8	5.31	80.7	7.21
BMI, kg m^−2^	24.5	2.53	25.1	2.64	25.1	2.70	24.3	2.76	26.1	2.03
Males, n	9		7		8		9		9	
Females, n	6		8		7		6		6	
Both	15		15		15		15		15	
Days after stroke	5	0.00	5	0.00	5	0.00	5	0.00	5	0.00
RH, n,%	3	14%	3	14%	7	33%	5	24%	3	14%
LH, n,%	11	28%	10	25%	5	13%	6	15%	8	20%
Cerebellar, n,%	3	7%	2	14%	3	21%	4	29%	4	29%
Smoking, n,%	11	73%	9	60%	13	87%	9	60%	6	40%
Alcohol, n,%	4	27%	4	27%	4	27%	2	13%	6	40%
mRS, median, IQR	3.0	1.0	4.0	1.0	4.0	1.0	4.0	1.0	3.0	1.0
										
Barthel index	64.0	8.28	62.7	7.99	61.8	8.95	65.7	9.04	61.7	9.57
Berg balance scale	19.9	2.43	20.8	3.14	20.7	4.20	22.1	2.43	23.2	4.26
6 MWT, m	119.7	15.86	114.3	15.10	111.7	13.18	122.7	24.85	116.0	13.12
6 MWT robot, m	232.7	33.69	241.3	35.63	233.3	36.38	230.0	39.10	226.7	36.97
10 m walk, m s^−1^	0.90	0.15	0.88	0.14	0.80	0.11	0.81	0.12	0.81	0.11
COP, cm s^−1^	21.1	1.78	20.4	1.99	21.1	1.78	20.6	2.07	21.5	2.04

CON, physical therapy control group; ROB, walking training while wearing a robot; EXE1, exergaming once a day; EXE2, exergaming twice a day; EXE1+ROB, one session each of walking training while wearing a robot and exergaming; Values in the $\bar{X}$ columns denote Mean or frequency, n; Values in the ±SD, % columns denote Standard Deviation or percent; BMI, body mass index; M, Males; F, Females; RH, right hemisphere stroke; LH, left hemisphere stroke; Cerebellar, cerebellar stroke; Alcohol, 1–3 drinks per day; mRS, Modified Rankin Scale, range: 0 (healthy) to 6 (death); IQR, interquartile range; 6 MWT, six-minute walk test; 6 MWT robot, six-minute walk test with robot; COP, center of pressure velocity.

**Table 2:** Post-hoc analysis of the between-group differences after the interventions.

Contrast	BBS	6 MWT No Robot	10 m	COP	6 MWT Robot
EXE1 vs. CON	4.7 (1.8 to 7.5); *p* = 0.001	119 (76 to 163); *p* = 0.001	0.15 (−0.021 to 0.320); *p* = 0.1126	−2.14 (−3.61 to −0.68); *p* = 0.001	63 (22 to 103); *p* = 0.001
EXE2 vs. CON	9.7 (6.8 to 12.5); *p* = 0.001	107 (63 to 150); *p* = 0.001	0.20 (0.030 to 0.370); *p* = 0.013	−1.55 (−3.00 to −0.11); *p* = 0.029	78 (37 to 118); *p* = 0.001
ROB vs. CON	3.7 (0.92 to 6.5); *p* = 0.004	64 (20 to 107); *p* = 0.001	0.22 (0.050 to 0.384); *p* = 0.005	−2.21 (−3.66 to −0.75); *p* = 0.001	99 (59 to 139); *p* = 0.001
EXE2 vs. EXE1	5.0 (2.2 to 7.9); *p* = 0.001	−13 (−57 to 32); *p* = 0.932	0.05 (−0.117 to 0.218); *p* = 0.916	0.59 (−0.86 to 2.04); *p* = 0.783	15 (−25 to 56); *p* = 0.829
ROB vs. EXE1	−0.9 (−3.8 to 1.9); *p* = 0.885	−56 (−100 to −12); *p* = 0.006	0.07 (−0.105 to 0.240); *p* = 0.809	−0.06 (−1.52 to 1.39); *p* = 0.999	36 (−4 to 77); *p* = 0.100
ROB vs. EXE2	−5.9 (−8.8 to −3.1); *p* = 0.001	−43 (−86 to 0); *p* = 0.048	0.02 (−0.154 to 0.188); *p* = 0.999	−0.66 (−2.10 to 0.79); *p* = 0.710	21 (−19 to 61); *p* = 0.578
EXE1+ROB vs. CON	5.9 (3.0 to 8.9); *p* = 0.001	155 (111 to 198); *p* = 0.001	0.30 (0.123 to 0.467); *p* = 0.001	−2.44 (−3.93 to −0.95); *p* = 0.001	157 (116 to 198); *p* = 0.001
EXE1+ROB vs. EXE1	1.3 (−1.6 to 4.2); *p* = 0.718	35 (−8 to 78); *p* = 0.158	0.15 (−0.0218 to 0.312); *p* = 0.118	−0.29 (−1.74 to 1.15); *p* = 0.980	94 (54 to 135); *p* = 0.001
EXE1+ROB vs. EXE2	−3.7 (−6.6 to −0.9); *p* = 0.005	48 (4 to 92); *p* = 0.026	0.10 (−0.074 to 0.264); *p* = 0.518	−0.88 (−2.35 to 0.59); *p* = 0.451	79 (39 to 120); *p* = 0.001
EXE1+ROB vs. ROB	2.2 (−0.7 to 5.2); *p* = 0.232	91 (47 to 135); *p* = 0.001	0.08 (−0.096 to 0.252); *p* = 0.717	−0.23 (−1.70 to 1.25); *p* = 0.993	58 (17 to 99); *p* = 0.002

Values are mean (95% confidence interval) of the between-group differences computed as the first vs. the second interventions listed (e.g., EXE1 minus CON) and the associated *p*-value; CON, physical therapy, standard-care control group; ROB, walking training while wearing a robot; EXE1, exergaming once a day; EXE2, one session of exergaming in the morning and one session of exergaming in the afternoon of the same day; EXE1+ROB, one session of walking training while wearing a robot in the morning and one session of exergaming in the afternoon of the same day; BBS, Berg balance scale scores; 6 MWT, six-minute walk test, m; 6 MWT robot, six-minute walk test with robot, m; 10 m, maximal walking speed in 10 m, m s^−1^; COP, center of pressure velocity, cm s^−1^.

**Table 3:** Post-hoc analysis of the between-group differences after detraining.

Contrast	BBS	6 MWT No Robot	10m	COP	6 MWT Robot
EXE1 vs. CON	8.8 (5.2 to 12.4); *p* = 0.001	155 (105 to 204); *p* = 0.001	0.10 (−0.103 to 0.311); *p* = 0.623	−2.69 (−4.36 to −1.03); *p* = 0.001	66 (9 to 123); *p* = 0.014
EXE2 vs. CON	10.8 (7.2 to 14.4); *p* = 0.001	130 (81 to 180); *p* = 0.001	0.19 (−0.017 to 0.395); *p* = 0.087	−1.85 (−3.50 to 0.19); *p* = 0.02	72 (15 to 128); *p* = 0.006
ROB vs. CON	3.8 (0.2 to 7.4); *p* = 0.03	73 (24 to 122); *p* = 0.001	0.23 (0.027 to 0.432); *p* = 0.019	−2.82 (−4.48 to −1.16); *p* = 0.001	92 (36 to 148); *p* = 0.001
EXE2 vs EXE1	2.0 (−1.6 to 5.6); *p* = 0.529	−25 (−75 to 26); *p* = 0.651	0.085 (−0.118 to 0.288); *p* = 0.769	0.84 (−0.81 to 2.50); *p* = 0.612	5 (−51 to 62); *p* = 0.999
ROB vs. EXE1	−4.9 (−8.6 to −1.4); *p* = 0.002	−82 (−132 to −32); *p* = 0.001	0.13 (−0.084 to 0.335); *p* = 0.453	−0.12 (−1.79 to 1.54); *p* = 0.999	26 (−31 to 83); *p* = 0.706
ROB vs. EXE2	−6.9 (−10.6 to −3.4); *p* = 0.001	−57 (−106 to −8); *p* = 0.014	0.041 (−0.166 to 0.248); *p* = 0.981	−0.97 (−2.62 to 0.69); *p* = 0.477	20 (−36 to 76); *p* = 0.849
EXE1+ROB vs. CON	6.4 (2.7 to 10.0); *p* = 0.001	153 (103 to 203); *p* = 0.001	0.289 (0.078 to 0.495); *p* = 0.002	−2.51 (−4.21 to −0.81); *p* = 0.001	143 (85 to 200); *p* = 0.001
EXE1+ROB vs. EXE1	−2.5 (−6.1 to 1.2); *p* = 0.335	−2.5 (−51 to 47); *p* = 0.999	0.182 (−0.020 to 0.385); *p* = 0.099	0.18 (−1.47 to 1.84); *p* = 0.998	77 (20 to 133); *p* = 0.003
EXE1+ROB vs. EXE2	−4.5 (−8.1 to −0.9); *p* = 0.008	22 (−28 to 72); *p* = 0.721	0.098 (−0.107 to 0.302); *p* = 0.669	−0.66 (−2.34 to 1.02); *p* = 0.805	71 (14 to 128); *p* = 0.007
EXE1+ROB vs. ROB	2.5 (−1.2 to 6.3); *p* = 0.338	79 (29 to 130); *p* = 0.001	0.057 (−0.154 to 0.267); *p* = 0.942	0.31 (−1.38 to 1.99); *p* = 0.986	51 (−7 to 108); *p* = 0.110

Values are mean (95% confidence interval) of the between-group differences computed as the first vs. the second interventions listed (e.g., EXE1 minus CON) and the associated *p*-value; CON, physical therapy control group; ROB, walking training while wearing a robot; EXE1, exergaming once a day; EXE2, one session of exergaming in the morning and one session of exergaming in the afternoon of the same day; EXE1+ROB, one session of walking training while wearing a robot in the morning and one session of exergaming in the afternoon of the same day; BBS, Berg balance scale scores; 6 MWT, six-minute walk test, m; 6 MWT robot, six-minute walk test with robot, m; 10 m, maximal walking speed in 10 m, m s^−1^; COP, center of pressure velocity, cm s^−1^.

## Data Availability

All data requests should be submitted to the corresponding author for consideration. Access to anonymized data may be granted following review.

## Abbreviations

6 MWT	6-min walk test
6MWTrobot	6-min walk test while wearing the robot
BI	Barthel Index
BBS	Berg balance scale
BMI	body mass index
BP	blood pressure
CI	confidence interval
CON	control group receiving standard physical therapy only
COP	center of pressure
CT	computed tomography
d	Cohen’s effect size
EEG	electroencephalography
EQ5-VAS	100 mm visual analog measure of health-related quality of life
EXE1	1 exergaming session once daily
EXE2	2 exergaming sessions twice daily
EXE1+ROB	exergaming and robot-assisted walking training
HR	heart rate
IQR	interquartile range
LH	left hemisphere stroke
mm Hg	millimeter of mercury
MMSE	mini-mental state examination
MRI	magnetic resonance imaging
mRS	modified Rankin Scale
pη^2^	partial eta squared
PwS	persons with stroke
RH	right hemisphere stroke
rHR	resting heart rate
ROB	robot-assisted walking training
